# The cultural sustainability in English as foreign language textbooks: Investigating the cultural representations in English language textbooks in China for senior middle school students

**DOI:** 10.3389/fpsyg.2022.944381

**Published:** 2022-10-05

**Authors:** Jun Lu, Yanhong Liu, Lin An, Ying Zhang

**Affiliations:** ^1^School of Economics and Management, Yanshan University, Qinhuangdao, China; ^2^School of Foreign Studies, Yanshan University, Qinhuangdao, China; ^3^Shanghai Center for Research in English Language Education, Shanghai International Studies University, Shanghai, China

**Keywords:** cultural sustainability, sustainability education, EFL textbooks, cultural representation, world Englishes

## Abstract

In the age of globalization, studies of cultural representations in foreign language textbooks take account of cultural sustainability. This article reports on a case study of cultural representations in two English language textbook series that are widely used in senior high schools in China. The study investigated whether the cultural representations in these textbooks contribute to the development of local cultural sustainability. Content analysis was employed to examine the texts used in these textbooks, with references to the synergy of the theoretical frameworks of cultural sustainability and world Englishes. The study results indicate that there is an imbalanced cultural representation with respect to the categories of international cultures and national culture in these textbooks. The results point to the cultures of the inner circle countries (BANA: Britain, Australasia, and North America) being predominant, while the representation of Chinese culture has a low profile. As these textbooks are an important learning and teaching resource in China, they have the potential to play a significant role in influencing learners’ worldviews as they develop their understanding of different cultures. The imbalanced presentation of culture may in turn lead to a biased worldview where learners, rather appreciating cultural diversity, may instead discriminate against certain cultures. The implications for redressing the imbalance in cultural representation and cultural sustainability are discussed.

## Introduction

Globalization is a multidimensional concept that cuts across a number of social sectors, such as economic sector, politics, and culture. Some globalization scholars (e.g., Robertson, 1992; Holton, 1998; Hopper, 2007) argue that the final stage of the process of globalization will achieve “de-territorialization” (Hopper, 2007, p. 48) with national borders erased, resulting in an homogenization that emphasizes global uniformity and ubiquity (Tomlinson, 1999).

To provide an example, with globalization impacting the education sector, in countries such as South Korea, “the model of English as a foreign language is standard North American, with cultural representations generally based on the life of North Americans or, in a few cases, the lives of the Britons and Australians” (Yim, 2003, p. 71). In this situation, globalization can be seen to affect the field of language and culture, and in particular English as foreign language (EFL)/English as second language (ESL) textbooks because of the cultural imperative in language learning (Liu, 2017). Similarly, some studies in the field of TESOL suggest that a large proportion of English language teaching (ELT) textbooks promote the culture of inner circle countries, particularly that of the United States, thus marginalizing, or neglecting, local cultures and/or those of outer circle and expanding circle countries (Yuen, 2011; Su, 2016; Liu, 2017; Sun and Kwon, 2020).

Against this background, concepts of cultural sustainability, cultural identity, and cultural continuity in education have recently received considerable attention. The concepts of cultural sustainability and sustainability education are aspects of sustainable development introduced by the World Commission on Environment and Development in 1997. Sustainability development has rich connotations and dimensions, and social sector and educational programs are indispensable parts to sustainability development (Soini and Birkeland, 2014). Cultural sustainability thus becomes one of the most significant discussions in the field of sustainability development.

Regarding the concept and practices of cultural sustainability, some researchers have carried out transdisciplinary work with an interest in exploring the concept of cultural sustainability and developed a three-role approach to defining cultural sustainability: (1) the “culture for sustainable development” approach regards culture as the “glue” that combines the ecological, social, and economic pillars of sustainable development (Dessein et al., 2015); (2) the “culture as sustainable development” approach views culture as the central part of sustainability (Dessein et al., 2015); and (3) the “culture in sustainable development” approach sees culture as having a separate and independent role in sustainable development, as a so-called fourth pillar in addition to those of ecological sustainability, economic sustainability, and social sustainability (Laine, 2016, pp. 52, 53).

Previous studies have explored the connotations and dimensions of cultural sustainability (e.g., Soini and Birkeland, 2014; Dessein et al., 2015; Laine, 2016). The three-role approach emphasizes different roles and significance of cultural sustainability from different perspectives (Laine, 2016). Collectively, these studies outline a critical role of cultural sustainability. The present study concurs with the study by Soini and Birkeland (2014), who argue that cultural change in the context of globalization is inevitable, but this should happen in a sustainable way. The primary question that needs to be considered is how the change can take place without damaging local cultural continuity, cultural identity, and cultural capital.

Education for Sustainable Development has triggered critical reflection on the cultural sustainability in foreign language education (Liu et al., 2015). While issues and uncertainties relating to cultural sustainability have emerged in ELT textbooks, they have not been sufficiently discussed across various contexts worldwide. The present study attempts to explore cultural representations that appear in two series of ELT textbooks used by senior high school students in China, with the intention of deepening our understanding of cultural sustainability in the language textbooks in question. In this article, the following research questions are addressed:

(1) What are the cultural representations in the two series of Chinese ELT textbooks?
(2) What role does these textbooks play in local cultural sustainability?

## Literature review

### The cultural imperative in language teaching

There are a large number of studies that describe the role of culture in language learning, especially within the domain of EFL teaching and learning. The notion of culture in Western academia has long been debated, resulting in defining the notion of culture being presented as a “perilous idea” (Wierzbicka, 1997, p. 17) and one of notorious difficulties (Williams, 1983) because the reason for culture being such a contested concept due to its intricate historical development and its use as a casual explanation of individual behavior (Atkinson, 2015).

When the concept of culture is seen from an academic perspective, there are three broad categorizations of the concept:

(i) Culture as an independent and abstract noun which describes a general process of intellectual, spiritual, and aesthetic development; (ii) the independent noun, whether used generally or specifically, which indicates a particular way of life, whether of a people, a period, or a group; and (iii) the independent and abstract noun which describes the works and practices of intellectual, especially artistic activity (e.g., music, literature, painting and sculpture, and theater and film) (Williams, 1976, p. 80).

According to Williams (1976), culture consists of elements of spiritual development, the way of life and the products of certain cultural activities. Seen from this perspective, culture plays an overarching role in education in terms of the development of individuals and groups of individuals (Kumaravadivelu, 2008). The imperative role can be found in the traditions of Europe and the United States and was identified in the National Standards in Foreign Language Education Project (1996). It became apparent that “culture formed the context in which all the other goal areas played out, this is to say, that culture became integrated with and integral to language learning” (Phillips, 2003, p. 163).

After some years of commitment to culture in foreign language education, the National Standards in Foreign Language Education Project (1996) in the United States presented a seminal and comprehensive document *Standards for foreign language learning: Preparing for the 21st century* (*Standards* hereafter) for the purpose of providing guidance to the language education profession. This document lists five major goal areas, which are referred to as the five C’s of foreign language education: Communication, Cultures, Connections, Comparisons, and Communities (Lafayette, 2003), with the further delineation of 11 standards; four of these standards directly address the meaning of culture in the education context:

Standard 2.1 Students demonstrate an understanding of the relationship between the practices and perspectives of the culture studied.
Standard 2.2 Students demonstrate an understanding of the relationship between the products and perspectives of the culture studied.
Standard 3.2 Students acquire information and recognize the distinctive viewpoints that are only available through the foreign language and its cultures.
Standard 4.2 Students demonstrate understanding of the concept of culture through comparisons of the cultures studied and their own (Lafayette, 2003, p. 58).

The guidelines of *Standards* are proposed for use in the foreign language study, with the rationale that foreign language learners need to develop the ability to understand the relationships between practices, perspectives, and products. Furthermore, these guidelines encourage the development of both the ability to recognize “the distinctive viewpoints through the foreign language and its cultures” and the ability to understand “the concept of culture through comparisons of the cultures studied and their own” (Lafayette, 2003, p. 58). Seen from this perspective, foreign language learning materials are expected to demonstrate and represent different cultures, both native and foreign cultures, to facilitate learners in developing the aforementioned abilities.

### Foreign language textbook studies

Although language textbooks play an indispensable part in foreign language education, particularly in outer circle and expanding circle countries, the study of language textbooks has attracted little attention. The 1990s marked a turning point for the role of culture in the field of foreign language education, with the appearance of scholarly works, Byram (1989), Kramsch (1993), and among others. Byram (1989) posited that the inseparable relationship between language and culture is reflected in the aims of foreign language education: the development of communicative competence for use in situations that students might expect to encounter, the development of an awareness of the nature of language and language learning, and the development of insights into foreign culture and positive attitudes toward foreigners. Kramsch (1993) later pushed for an understanding of culture that went beyond language learning to a higher level, formulating the concept of culture as an independent aspect of general education.

White (1998) argued that textbooks serve three aspects of language: (1) linguistic components—grammar, vocabulary, and language skills; (2) pedagogy—beliefs and practices about teaching and learning; and (3) content—situational contexts and topics. The second and third components of the previously described content are closely related to culture. Venezky (1992) thus argued that textbooks are both cultural and curricular artifacts. This is to say that intended curriculum and cultural values are either transmitted explicitly or implicitly *via* the textbooks used (Nozaki et al., 2005). Given the imperative role of culture in sustainability development, particularly in language education sustainability, a concomitant interest has emerged in the investigation of the cultural constellations of local culture and other world cultures in EFL textbooks (e.g., Yuen, 2011; Liu et al., 2015).

Given the imperative of culture in language learning, Kachru (1992) used a circle analogy, in which he placed countries in three circles: the inner circle, outer circle, and expanding circle, with references to the historical, sociolinguistic, and literary contexts. The inner circle refers to the traditional cultural and linguistic bases of English, with English being learned as ESL in inner circle countries, and as EFL in outer circle countries. There are problems, however, with EFL/ESL textbooks worldwide in non-English-speaking countries or regions. Textbooks play a significant role in language education, particularly in “expanding circle” countries (Kachru, 1992), including China, Japan, and South Korea, where English is taught as a second language or English as foreign language is taught (Liu, 2017). In these countries, because textbooks are “key resources for formal English education in that they are used directly for both everyday school-wide teaching and nation-wide high-stakes tests” (Song, 2013, p. 383), they inevitably have a significant influence on cultural sustainability.

Several studies, originating in a number of countries, have investigated English language textbooks, with reference to issues of globalization, localization, cultural identity, and cultural sustainability. For example, studies from Japan (e.g., Matsuda, 2002; Otlowski, 2003; Yamanaka, 2006; Schneer, 2007), South Korea (e.g., Yim, 2003, 2007; Song, 2013; Lee, 2014), Thailand (Suaysuwan and Kapitzke, 2005), Canada (Ilieva, 2000; Gulliver, 2010), Hungary (Weninger and Kiss, 2013), Romania (Camase, 2009), Colombia (Moss et al., 2015), and Singapore (Curdt-Christiansen, 2015) all have highlighted major problems, such as cultural aphasia in foreign language textbooks.

In South Korea, Lee (1999), in a study of culture representation in Korean language textbooks, analyzed textbooks across four different periods: the pre-colonial, colonial, neo-colonial, and globalized periods. The findings revealed that the values of dominant interest holders—Japan and the United States—are reflected in textbooks and that local Korean cultural and historical dimensions were either omitted or misrepresented.

Singaporean researchers Gupta and Lee (1990) and Curdt-Christiansen (2015) have provided some insights into English textbooks used in the multiracial and multilingual context of Singapore. Curdt-Christiansen (2015) examined two sets of English language instructional materials for Singaporean primary schools and found that there was an absence of local children’s literate identities. Instead, children were depicted in stories as the idealized model of Western middle-class children. With little representation of “self,” the textbooks cannot provide relevant cultural capital and language to users to communicate with the world. Similarly, Tajeddin and Teimournezhad (2015) explored ELT textbooks and found that “no reference was made to the learners’ source culture” (p. 188). Likewise, in Thailand, Suaysuwan and Kapitzke (2005) reported that “textbooks privilege Western values and ideas, such as Western-style housing, food, characters in the stories, while indigenous culture was excluded from textbooks” (p. 92).

In Japan, several studies have investigated the cultural content in textbooks (e.g., Hino, 1988; Koike and Tanaka, 1995; Matsuda, 2002; Yamanaka, 2006). Hino was the first researcher in Japan to discuss how to maintain national and cultural identity in the context where English both dominated and was advocated as an international language. By examining the cultural components of English textbooks in Japan during the past 120 years, Hino was able to identify both the impact of the chauvinism of native speakers on the Japanese population and the nationalism of non-native speakers that impacted the English language education in Japan. This project covering textbooks used during a 120-year period was divided into five periods, enabling Hino to claim that Japanese attitudes toward English education, in textbooks, fluctuated from “blindly venerat[ing] the Anglo-American cultures” to “total exclusion of foreign cultures and extreme appreciation of the Japanese culture,” to “exclusively Anglo-American culture” (p. 309). Hino concluded that neither the jingoism of native English speakers nor the radical and narrow nationalism of non-native speakers was acceptable. Furthermore, he asserted that these attitudes were not incompatible with the concept of English as an international language.

In China, English language textbook study mainly concentrates on investigating the linguistic characteristics. Some academics conducted systematic studies on the lexical coverage, text readability, and frequency of re-occurrence of Chinese EFL textbooks (e.g., Chen, 2011; Liu and Liu, 2011; Liu and Zhang, 2015; Zhang and Liu, 2015). Although these research studies created or built a foundation for future empirical studies, also providing potentially useful and practical support for ELT, they all focused exclusively on the first level, which involved the investigation of linguistic items, such as vocabulary size, coverage, frequency, distribution, and collocation. The non-linguistic aspects of textbooks —for example, cultural representation and ideology underpinnings—are yet to be given due attention.

Some attempts to examine cultural representations and ideological underpinnings in EFL textbooks, although relatively recent, have been made. Su (2016) found that English language textbooks used in Taiwan—textbooks in which American and British cultures predominate—rarely contain references to Chinese culture. Yuen (2011), aiming to examine the extent to which the textbooks include the cultures of the world, analyzed the representation of foreign cultures in Hong Kong EFL textbooks used in secondary schools. A relatively comprehensive account involved counting the references to products (different aspects of culture), perspectives (thoughts), practices (communication), and persons (different people). Yuen found that the cultures of inner circle countries are more apparent in Hong Kong EFL textbooks.

Liu et al., 2022a,b built a corpus of university EFL textbooks and explored the cultural representations, ideological underpinnings, and the representation of Western religious beliefs in these textbooks. These researchers posited that the dominance of the monocultural representations, mainly of Anglo-American cultures, is problematic in an increasingly multicultural world.

The aforementioned studies are mainly on EFL university textbooks; however, the status of textbooks for middle school students has been rarely explored. According to the latest available The Ministry of Education of People’s Republic China (2017), China has the largest population of EFL learners in higher education institutions in the world. The number of middle school students is much larger than that of university students because of the college/university entrance examination. Foreign language learning and teaching in China mainly rely on textbooks. These textbooks are the principle medium through which learners acquire foreign linguistic skills and learn about the world outside. Therefore, the cultural landscape in these textbooks has the potential to play a significant role in influencing learners’ cultural sustainability and the formation of their worldviews. This potential also influences the formation of their cultural continuity, cultural identity, and cultural capital, and development that also fosters their understanding of different cultures.

In summary, the aforementioned literature review informed the focus of this study, which has the purpose of examining cultural representations in two sets of English language textbooks for senior middle school students in China, with an interest in better understanding the impact of such cultural representations on the cultural sustainability of these students. The findings may have future implications for the development of EFL textbooks not only in China but also worldwide.

## Methodology

### Theoretical framework

To address the research questions in relation to the cultural content in the textbooks, this study was constructed using a synergy of the theoretical frameworks of Laine’s (2016) cultural sustainability and Kachru’s (1992) world Englishes. Laine (2016) defined the concept of cultural sustainability in education and introduced three themes of cultural sustainability: local culture, national culture, and global culture. According to Laine (2016), local culture refers to “[l]ocal environment and culture as well as local traditions”; national culture refers to “[n]ational culture and cultural heritage”; and global culture refers to “[i]nternationality and multiculturalism” (p. 56). This theoretical framework informs the present study with regard to the categorization of cultural presentations in the textbooks. In our study, the theme of local culture has to be merged into the theme of national culture in response to the reality that the textbooks in question are developed at national level, meaning the representation of local culture in these texts is not evident. Laine’s (2016) theoretical framework provides detailed subthemes of the three dimensions of culture, with a description of each subtheme at both local and national levels. However, the subthemes and their descriptions at international level are not sufficient in their complexity to produce the categorization of international cultures. Kachru’s (1992) stratification of English provides supplementary support for this weakness in Lane’s framework.

Kachru’s (1992) stratification of English closely connects foreign language learning and culture. This is because words and their meanings are linked to cultural context, wherever language is used. Cultural representation in this study is viewed as being closely related to English language study because of the imperative of culture in language learning. Kachru’s analogy using the three circles of countries (the inner circle, outer circle, and expanding circle) makes reference to the historical, sociolinguistic, and literary contexts and, as such, is regarded as an appropriate stratification of cultural representation for our study. This linguistic geography will better inform the analysis of cultural representations in this study than those representations that are defined according to geographical stratifications (e.g., Yuen, 2011). Kachru’s stratification is as follows:

The current sociolinguistic profile of English may be viewed in terms of three concentric circles. … The Inner-circle refers to the traditional cultural and linguistic bases of English. The Outer-circle represents the institutionalized non-native varieties (ESL) in the regions that have passed through extended periods of colonization. The Expanding-circle includes the regions where the performance varieties of the language are used essentially in EFL contexts (Kachru, 1992, pp. 356, 357).

This stratification is further illustrated by (Kachru, 1992, p. 3).

• The “expanding circle”:

for example, China, Egypt, Indonesia, Israel, Japan, Korea, Nepal, Saudi Arabia, the USSR, and Zimbabwe.

• The “outer circle”:

for example, Bangladesh, Ghana, India, Kenya, Malaysia, Nigeria, Pakistan, the Philippines, Singapore, Sri Lanka, Tanzania, and Zambia.

• The “inner circle”:

The United States, the United Kingdom, Canada, Australia, and New Zealand.

In addition, there are three other categories: mixing cultures, common culture, and unspecified culture, which are added to the general categorization. These additional categories and descriptions are as follows:

• Mixing cultures are presented in texts that introduce, mix, or compare cultures of different countries;
• Common culture are included in texts that discuss some universal topics, such as global warming and the Internet; and
• Unspecified culture refers to cultures in texts for which there is no clear indication of their origins.

### Data collection

The content analysis approach, used in extant studies of language textbooks (e.g., Ndura, 2004; Schneer, 2007; Tajeddin and Teimournezhad, 2015), was employed to identify the cultures represented in the textbooks. The textbook series, New Senior English for China (2004) and English (2019) for senior middle school students, were analyzed in this study because they are core teaching materials in China. These English textbooks were published by the People’s Education Press (PEP) administered by the Ministry of Education of China, the main teaching material publishing agency in the education sector. The *New Senior English for China* (*NSEC*) comprises five books, and *English* comprises three books.

Each unit of the two textbook series contains sections, such as “Listening and Speaking,” “Reading and Thinking,” “Listening and Talking,” “Reading for Writing,” “Project,” and “Video Time.” The sections involving “Listening,” “Watching Videos,” or “Project” may not be used by some teachers at some schools because of the lack of teaching facilities or because of the way the entrance examination is oriented toward the teaching curriculum. Given this situation, we have focused our study on the reading passages, which contain the foreign cultural components represented in these textbooks.

## Data analysis

A content analysis of the textbook series was conducted. The textbook texts relating to cultures of different subthemes were abstracted. Textbook texts are identified and categorized according to how they reference the evidence of specific cultural content. In most of the texts, the connections with the origins of cultures are explicitly indicated. For example, the cultural origins in the texts “Winter Carnival in Quebec,” “The Story of Atlanta,” “Terracotta Army,” and “Beautiful Ireland and Its Traditions” can be easily identified and categorized. However, sometimes, the cultural origins are not evident in the title of the text and are instead indicated in the text itself. For example, in a narrative story “The Cab Ride I’ll Never Forget,” the author relates a touching story about how, as a taxi driver, he was sent to take an old lady to a hospice. The context where the story occurred is not explicitly indicated, but rather expressed with the words “I drove a cab for a living. It was a cowboy’s life…” and “She was wearing a print dress and a pillbox hat with a veil pinned on it, like somebody out of a 1940s movie,” which suggests the cultural origin of this text—would be the United States.

In the aforementioned cases, the names of original cultures were used to code the data and identify units, which would later be counted in the final stage of the data analysis. Specifically, the products (e.g., books, tools, foods, laws, music, and games), practices (e.g., patterns of social interactions), perspectives (e.g., meanings, attitudes, values, and ideas), or persons (e.g., the figures of some cultures) associated with specific country were identified as being phenomena that were representative of their particular culture.

**FIGURE 1:**
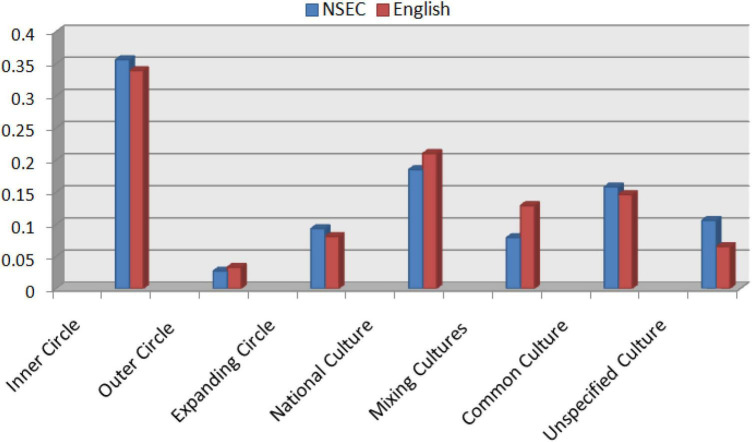
Cultures in textbook series *NSCE* and *English*.

The representations of cultures in the textbooks studied were identified, the data for which are shown in Figure 1. The specific classifications are given in Tables 1, 2. The resulting cultural representations show an imbalance in the cultural representation of different countries.

**TABLE 1:** Cultural representations in textbook series *NSEC*.

Texts	International cultures				National culture	Common culture	Unspecified culture
	
	Inner circle	Outer circle	Expanding circle	Mixing cultures			
Book1-U1	1		1		1		
Book1-U2	3						
Book1-U3			1		2		
Book1-U4	1				2		
Book1-U5	1	2					
Book2-U1			1	1	1		
Book2-U2			1	2			
Book2-U3						3	
Book2-U4					1	2	
Book2-U5	2						1
Book3-U1	1			1	1		
Book3-U2	1				2		
Book3-U3	3						
Book3-U4						2	1
Book3-U5	3						
Book4-U1	2				1		
Book4-U2					2	1	
Book4-U3	3						
Book4-U4				2		1	
Book4-U5	1		1				1
Book5-U1	1		2				
Book5-U2	3						
Book5-U3							3
Book5-U4						1	2
Book5-U5	1					2	

**TABLE 2:** Cultural representations in textbook series *English*.

	International cultures				National culture	Common culture	Unspecified culture
	
Texts	Inner circle	Outer circle	Expanding circle	Mixing cultures			
Welcome	1	1			1		
Book1-U1				1	1	1	1
Book1-U2			2		1		1
Book1-U3	2			1	1	1	
Book1-U4	1				1	1	
Book1-U5	1				2	1	
Book2-U1			1	2	1		
Book2-U2				1	2	1	
Book2-U3	1			1		1	1
Book2-U4	1	1	2				
Book2-U5	3			1			
Book3-U1	2			1	1		
Book3-U2	1				2		1
Book3-U3	3						
Book3-U4	1					3	
Book3-U5	4						

## Results

### International cultures

#### Inner-circle cultures

Figure 1 reveals that the cultures of inner circle countries are overwhelmingly prominent in both sets of textbooks. A close look at the configuration of these cultures in each textbook shows that content from these cultures makes up 35% of the cultural evidence in *NSEC* and 34% of the cultural evidence in *English* series. A detailed distribution of the cultures indicates that inner circle cultures appear in most units of the sets of the textbooks (see Tables 1, 2). In some units, the content of all three texts contains references to the cultures of inner circle countries, for example, in *NSEC*, Book 1-Unit 2, Book 3-Unit 3, Book 3-Unit 5, Book 4-Unit 3, and Book 5-Unit 2. In some units, three texts of one unit contain references to the culture of a specific country. For example, *NSEC* Book 3-Unit 5, Text A “A Trip on ‘The True North” introduces Canada by taking readers on a trip across the whole continent from west to east. See, for example, the following excerpt:

#### Excerpt 1

*You’re going to see some great scenery. Going eastward, you’ll pass mountains and thousands of lakes and forests, as well as wide rivers and large cities. Some people have the idea that you can cross Canada in less than 5 days, but they forget the fact that Canada is 5,500 km from coast to coast. Here in Vancouver, you’re in Canada’s warmest part. People say it is Canada’s most beautiful city, surrounded by mountains and the Pacific Ocean. Skiing in the Rocky Mountains and sailing in the harbor make Vancouver one of Canada’s most popular cities to live in. Its population is increasing rapidly. The coast north of Vancouver has some of the oldest and most beautiful forests in the world.*

Furthermore, Text B, “The True North’ from Toronto to Montreal,” focuses on the southern cities of Toronto and Montreal, and Text C “Iqaluit—The Frozen Town” refers to the culture of Inuit people who reside in the “farthest northeastern area of Canada, north of the Arctic Circle.”

#### Excerpt 2

…*Iqaluit, a town with a population of 6,000*… *It was two o’clock in the afternoon, but it was already dark, and all the houses shone with bright lights. Beth said, “Why is it so dark? It’s the middle of the day!” Simon replied, “It’s dark in the day because we are so far north. You should come in June. The sun shines all night in the north then. That’s why it’s called ‘The Land of the Midnight Sun”’. There were people on the streets and snowmobiles everywhere.*

As discussed previously, in the *NSEC* series, three texts in one unit all refer to the cultures of inner circle countries. This situation is replicated in the textbook series *English*, which was published 15 years later. The distribution of cultures in the textbook series *English*, as shown in Table 2, demonstrates that the cultures of inner circle countries predominate in three or four texts of one unit, for example, Book 2-Unit 5, Book 3-Unit 3, and Book 3-Unit 5. The cultural presentation in this case indicates, to some extent, that the scope of cultural sustainability in PEP textbooks has not changed. For example, in Book 2-Unit 5 and Book 3-Unit 3, the cultural representation features famous people from inner circle countries. In the *English* textbook series, the award-winning composer and conductor Eric Whitacre in “The Virtual Choir” and Ludwig van Beethoven in “Ludwig van Beethoven” are used as cultural icons representing inner circle cultures. The textbook series in our study have some characteristics in common with respect to their presentation of inner circle cultures.

#### Outer circle cultures

The cultures of outer circle countries are under-represented. There are only two texts that refer to such cultures in both the *NSEC* series and the *English* series. In the *NSEC* series, the two texts, “Elias’ Story” and “The Rest of Elias’ Story,” refer to the former leader of South Africa—Nelson Mandela, who fought for the freedom of the indigenous people of South Africa, for which he was prisoned for almost 30 years. He gave his life to helping his people to get the same rights as white people. These two texts begin with Elias’ narrative on Mandela’s great deeds:

#### Excerpt 3

*My name is Elias. I am a poor black worker in South Africa. The time when I first met Nelson Mandela was a very difficult period of my life. I was 12 years old. It was in 1952 and Mandela was the black lawyer to who I went for advice. He offered guidance to poor black people on their legal problems. He was generous with his time, for which I was grateful.*

In the textbook series *English*, two texts relate to outer circle cultures: “Student Profile—South Hill High School of South Africa” and “Beautiful Ireland and Its Traditions.” In the South African student’s profile, while few cultural elements are evident, there is mention of the student’s nationality. The text is as follows:

#### Excerpt 4

*I’m Thando Gowon. I’m 16 this year. I come from South Africa. I’m a Grade 10 student at South Hill High School. I look good, think fast, and play hard. You’ll never see me without a book or a pen. If I’m not in class, I’m either in the library or in the computer lab. At the weekends, I play computer games if I’m not busy studying. My dream is to start my own IT company.*

In another text with cultural evidence of life in Ireland, some unique aspects of Irish culture, including natural and humanistic features are evident in the following excerpt:

#### Excerpt 5

*Ireland’s beautiful countryside has always had a great influence on its people and traditions. The country has a long history of producing great writers and poets. Its beautiful countryside excites and inspires all, offering something for each of the senses*…*With all this beauty, it is not surprising that Ireland has developed strong traditions that include music, dancing, and dining*…*stop by a village pub and relax with a glass of wine or local beer*…

The aforementioned excerpt provides a relatively comprehensive presentation of Ireland’s culture and history. However, the representation of other outer circle cultures of this kind is insufficient.

The four texts relating to cultures of outer circle countries only focus on “people” (Elias and Thando Gowon of South Africa) and “practice” (sightseeing in Ireland’s countryside). The students’ profiles do give some basic introduction to their lives, but these texts do not provide a clear insight into lives of people in South Africa. Also, the description of Ireland’s beautiful countryside introduces some of the subthemes of cultural sustainability: “natural heritage,” “landscapes,” and “history appearing in the environment” (Laine, 2016). However, other aspects or subthemes such as cultural customs, cultural heritage, and awareness of history are absent.

#### Expanding circle cultures

The content referring to the cultures of expanding circle cultures is relatively limited: 9.3% in the *NSEC* series and 8.1% in the *English* series. In the textbook *English*, there are five texts referring to expanding circle cultures. Two texts are about life in Peru and introduce readers Peru’s history, geography, language, and people, with particular emphasis given to tourism experiences: “Amazon Rainforest Tour,” “Machu Picchu Tour,” “Cusco Tour,” and “Lake Titicaca Tour.” In spite of some sporadic references to people’s cultural practices in Peru, these texts mainly focus on cultural landscapes, especially Peru’s natural environment and ecosystem.

While the text “Machu Picchu Tour” highlights Peru’s ancient Architectural achievements—for example, the Incas’ dry-stone method of building; one of the other three texts contains two letters in which the authors share their travel experiences in Cairo and Athens, in which tourists mostly refer to the scenery and the local food. However, in the other texts “Samovar—the Special Teapot” and “From Problems to Solutions,” the texts explore the cultures in depth as follows:

#### Excerpt 6

…*special teapot—the samovar! It’s a traditional Russian water boiler that can also brew tea. It played a big part in Russian history and was a central part of Russian family life for a very long time*…*Almost 200 years ago, my great-grandmother’s great-grandmother was trying to survive yet another freezing Russian winter when she heard about a new invention called a “samovar,” which means “self-boiler” in Russian*…*The samovar tea-making process is quite special and has two stages. First, a teapot containing lots of tea leaves and a little water is placed on top of the samovar*…

This excerpt describes the cultural history of a Russian teapot, the “Samovar,” providing details about its invention, its integration into Russian family life, and its tea-making process, while at the same time highlighting its cultural symbolism: “The samovar symbolizes happiness, and that’s one thing that I want to keep in my family forever!” This is a typical text referring to the subtheme “Interaction between Generations” of cultural sustainability, which introduces stories that one generation will share with another through the special samovar teapot. This story contributes to cultural sustainability for reason that it portrays “learning from and appreciating one’s family” and “bringing together different generations and times.” As Laine (2016) indicates, “[c]ulturally sustainable development takes different generations into account” (p. 57).

The text “From Problems to Solutions” presents readers with a practical modern problem: How to build a new dam across the Nile to control floods, produce electricity, and supply water to more farmers in the area? Protests arose because water from the dam would likely damage a number of temples and destroy cultural relics that are an important part of Egypt’s cultural heritage. This problem was addressed, by moving the temples and other cultural sites to be effected by the flooding of the dammed area to a place where they would be safe after the Aswan Dam was completed. The relocation project was considered a great success, in particular because this was achieved through the united efforts of the international community. The text introduces the solution to the problem of preserving ancient cultural relics when needing to also allow for progress in the form of modern development.

In the textbook series *NSEC*, there are seven texts on the cultures of expanding circle countries, which differ from those in the textbook series of *English*. In *English*, the cultural representations of expanding circle countries mostly refer to beautiful scenery, while the cultural representations in the *NSEC* series tend to involve the presentation of literature and scientific achievements. For example, two texts that refer to literature highlight the importance of Anne’s Diary and the Greek story—The Story of Atlanta, while two texts refer to “Copernicus’ Revolutionary Theory” and “Finding the Solution,” introducing the great astronomer Nicolas Copernicus and the mathematician Leonhard Euler.

### National culture

The textbook series *NSEC* contains 13 texts (17.1%) that represent Chinese culture. The textbook series *English* also includes 13 texts (21%) with reference to Chinese culture, as shown in Table 3.

**TABLE 3:** Chinese culture in two textbook series.

Text-books	Text	Content	Text source
*NSEC*	A letter from Xiao Dong	A letter from a Chinese student of senior high school to his teacher	Book1-Unit1
	The dream and the plan	Some Chinese students’ bike trip—part I	Book1-Uint3
	A night in the mountains	Some Chinese students’ bike trip—part II	Book1-Uint3
	A night the earth didn’t sleep	An earthquake happened in China	Book1-Uint4
	A letter of invitation	An invitation of speaking competition winner to a Chinese city	Book1-Uint4
	Big Feng to the rescue	A Chinese man tries to protect the cultural relics in Tianjin.	Book2-Unit1
	The return of the Milu deer	The story of Miludeers return China from the United Kingdom.	Book2-Unit4
	A sad love story	An introduction of Chinese Valentine’s Day.	Book3-Unit1
	Come and eat here 1	The story of Chinese restaurant 1.	Book3-Unit2
	Come and eat here 2	The story of Chinese restaurant 2.	Book3-Unit2
	Why not carry on her good work?	The story of a Chinese doctor Lin Qiaozhi.	Book4-Unit1
	A pioneer for all people	An introduction of Chinese scientist Yuan Longping.	Book4-Unit2
	An early farmer pioneer	An introduction of Chinese farmer pioneer Jia Sixie.	Book4-Unit2
*English*	First impressions	Two senior high students’ first impressions of their schools.	Starter Unit
	A letter of advice	An adviser for teenagers expresses her advice about computer games to a Chinese boy.	Book1-Unit1
	A travel plan to Xi’an	Write to a friend about a travel plan in Xi’an, a Chinese city.	Book1-Unit2
	The night the earth didn’t sleep	An earthquake in Hebei Province of China.	Book1-Unit 4
	The Chinese writing system	The history of Chinese wiring system.	Book1-Unit 5
	Learning English	Chinese students’ problems with learning English from an online forum.	Book1-Unit 5
	Promoting culture through digital images	China’s ancient cultural heritage—Mogao Caves by recording and collecting its digital images.	Book2-Unit 1
	A day in the clouds	The protection of Tibetan antelopes.	Book2-Unit 2
	The strange tale of the Milu Deer	The history and protection of China’s Milu Deer.	Book2-Unit 2
	My amazing Naadam Experience	The experience of Naadam Festival in China’s Inner Mongolia Autonomous Region.	Book3-Unit 1
	Mother of Ten Thousand Babies	The story of a Chinese doctor Lin Qiaozhi.	Book3-Unit 2
	The five virtues	The five virtues in Chinese culture.	Book3-Unit 2

As shown in Table 3, the texts referring to Chinese culture involve engagement with several content areas, such as cultural icons (*NSEC*: Why not carry on her good work? A pioneer for all people, An early farmer pioneer; *English*: Mother of ten thousand babies), traditional cultural heritage (*NSEC*: Big Feng to the rescue, A sad love story; *English*: The Chinese writing system, Promoting culture through digital images, My amazing Naadam experience, The five virtues), environmental issues and natural features (*NSEC*: A night the earth didn’t sleep, The return of the Milu deer; *English*: The night the earth didn’t sleep, A day in the clouds, The strange tale of the Milu Deer), and students’ experiences in/out of schools (*NSEC*: A letter from Xiao Dong, The dream and the plan, A night in the mountains, A letter of invitation; *English*: First impressions, A letter of advice, A travel plan to Xi’an, Learning English).

Regarding Chinese culture, the two textbook series mainly include texts relating to students’ personal experiences and events, as well as some Chinese scenic spots and great historical figures. While these elements are aspects of Chinese culture, the connotation, in this case, is that culture is not limited to social interactions and personal experiences. According to National Standards in Foreign Language Education Project (1996), culture in foreign language education involves different aspects of culture, which can be termed “products,” “practices,” and “perspectives.” The texts in questions involve less in the way of cultural references relating to “perspectives” and “products.” The preservation and transmission of cultural heritage and traditions, such as thinking patterns, the products of music, and literature, are essential to cultural sustainability. Meanwhile, “from the standpoint of sustainable development, one should see examples from different cultures alongside with one’s identity and surroundings so that development could be globally sustainable” (Laine, 2016, p. 57).

### Mixing cultures

There are six texts (7.9%) in the *NSEC* series containing cultural references to “mixing cultures,” and eight texts (12.9%) in the *English* series referring to “mixing cultures.” The details of these texts are as follows, respectively.

The texts in Table 4 introduce or compare the cultures of different countries in a single text. For example, “The open hand-a universal sign” in the *NSEC* series introduces the ways people greet each other in Japan, India, Muslim countries, and the Western world. Another example in the *English* textbook series is the text “World cultural heritage sites,” which presents the world’s cultural and natural heritages (as highlighted by UNESCO), such as the Taj Mahal in India, Angkor Wat in Cambodia, and Cologne Cathedral in Germany. These texts introduce cultures throughout the world that are thought to be of vital importance to human history and culture.

**TABLE 4:** Mixing cultures in the two textbooks series.

*NSEC*	*English*
A fact or an opinion	Teenage life around the world
An interview	Living legends
Three inspiring stories about the Olympic Games	New discoveries from the past
Festivals and celebrations	Urban wildlife
Communication: no problem?	E-learning
The open hand-a universal sign	Music scores in films
	Why do we celebrate festivals
	World cultural heritage sites

### Common culture and unspecified culture

The category “common culture and nationality unclear” accounts for 26% of the texts in the *NSEC* series and 21% in *English*. Texts on common culture and unspecified culture discuss some universal topics with cultural origins that are not clearly indicated, for example, science topics, such as “Is exploring space a waste of time and money” and “Homes on Mars,” or global trends and social issues, such as “The face-down generation” and “Online safety.” This category is not discussed in this article because the unspecified culture is irrelevant.

## Discussion

The interrelationship between culture and development was discussed during the UNESCO Decade of Culture and Development (1988–1997) (Soini and Birkeland, 2014). The right that people have access to their own language and culture is included in the UN Declaration of Human Rights. As (Laine, 2016, p. 63)) says, applying them to the present time, the “strengthening of one’s own identity and one’s own roots was considered a central development target in educational practices, along with appreciating, treasuring, upholding and maintaining one’s own culture and traditions.”

The cultural representations in English language textbooks differ in different expanding circle countries in different periods of history. The relatively low incidence of reference to Chinese culture in the Chinese textbook series differs from that found in South Korean EFL textbooks. Through content analysis of four South Korean EFL textbooks, Song (2013) found that South Korean EFL textbooks tend to highlight South Korean culture with two textbooks showing South Korean dominance, while one had an equal number of South Korean and American characters. These findings are similar to those found in Yim’s (2003) study on South Korean EFL textbooks. Yim (2003, p. 178) found that South Korea cultural prevails in EFL textbooks where “the cultural content of these textbooks is designed with the priority of helping Korean students become aware of their own cultural identity.”

While ideal cultural representation is unlikely to be achieved, an imbalance in cultural representation is problematic in terms of cultural sustainability because of the role culture plays in the education and development of individuals and groups of individuals (Kumaravadivelu, 2008). The dominance of particular cultures, for example, in particular inner circle cultures, leads to the marginalization of local culture and may hinder the development of cultural sustainability. For example, Hino’s (1988) investigation of English language textbooks in Japan reflected a concept of native-speakerism supremacy (Liu et al., 2022a). A similar issue concerns *innercirclism*, which is thought of as a persistent narrative in the recent exploration of multimodal discourse in ELT textbooks (Smith, 2021).

Sustainable development goals aim “[b]y 2030, [to]ensure that all learners acquire knowledge and skills needed to promote sustainable development, including, among others, through education for sustainable development and … global citizenship and appreciation of cultural diversity and culture’s contribution to sustainable development” (UNESCO, 2017, p. 8). First-language textbooks and foreign language textbooks are the main source of historical, sociolinguistic, and literary knowledge in language teaching and learning. Cultural information and cultural values may be conveyed in the form of dialog, descriptive texts, and interactive activities (Luke, 1988; Cunningsworth, 1995). The dominance of specific cultures may result in the erosion of other cultures, including the culture of the first language, and negatively influence cultural sustainability.

In addition, the dominance of cultures of inner circle countries may contribute to developing a sense of linguicism in EFL learners. The dominance of some cultures may lead to a situation where only specific cultures, values, and norms are recognized and accepted, while others are excluded, leading to a cultural fault line, which is not conducive to the building of cultural identity, and connect it to the cultural diversity in the global context toward sustainable development. Kramsch (1993) argued that understanding a foreign culture requires relating that culture to one’s own culture, and that cultural interconnection entails going through at least four steps of perceptions and reflections on the multifaceted nature of our cultural reality: C1 perception of self, C1 perception of others, C2 perception of self, and C2 perception of others with C1 indicating native culture and C2 target culture (C1: first-language culture; C2: foreign language culture). Kramsch (1993) argued that C1 and C2 are indispensable “in a process of (1) deconstruction of a target culture (C2) and a native culture (C1) to get a perception and connection and (2) reconstruction of C2 and C1 by making a reflection both on C2 and C1 along the ‘cultural faultline’ to establish a ‘sphere of interculturality”’ (Liu et al., 2022a, p. 3).

Seen from this theoretical ideal, an imbalanced cultural representation may hinder the process of intercultural understanding and learning. Marginalization of first-language culture may not give learners the opportunity to deconstruct, reflect, and reconstruct both native and target cultures. Furthermore, the high proportion of foreign cultures, mainly American and British cultures and the limited content of Chinese culture, in EFL textbooks, cannot provide learners with cultural aggregates of their own culture, which may result in cultural aphasia leading to a sense of cultural inequity or cultural inferiority.

Laine (2016) argued that a culturally sustainable education considers culture on the local, national, and global levels and supports the identity development process of the pupil (p. 64). An imbalance of cultural content, in particular of local and global cultures, may handicap the development of cultural sustainability, as well as the development of equal intercultural communication. Cultural sustainability has been emphasized as a fourth pillar of sustainability (Hawkes, 2001) because culture is considered a central aspect of the preservation of sociocultural patterns (Vallace et al., 2011). Therefore, cultural heritage, both local and global, as the cultural capital that has been inherited from previous generations can be transmitted to future generations to meet the changing needs of globalization.

## Limitations

With respect to the limitations of our study, the following points might merit further discussion. First, regarding the inclusiveness of cultural representation, the study focuses on texts of two series of textbooks. If more content, such as the text images, project, and classroom tasks, were included, the study might have produced more comprehensive results. Second, future studies might include teachers’ responses to text delivery and classroom instruction because teachers, as textbook users, may supplement relevant cultural content provided by the textbooks.

## Conclusion

The results of the content analysis of the two textbook series engaged in this study show an imbalanced cultural representation. The cultures of inner circle countries are predominant in the textbooks. Cultures from outer circle countries are least represented with only two texts in the *NSEC* and *English* textbook series. The content referring to cultures of expanding circle cultures, excluding China, are marginalized, which accounts for 8–9% in both series of textbooks. The representation of Chinese culture has a low profile compared with the cultures of the inner circle countries. The texts that make reference to the cultures of different countries are limited in both series of textbooks. The category of common culture and unspecified culture makes up 21% in the *English* series and 26% in the *NSEC* series. This finding is supported by Liu et al. (2022a) who found that in 10 sets of Chinese university English language textbooks, the texts representing Chinese cultures only account for 3% of the total cultural content evidenced in these texts.

A fully balanced cultural representation in terms of local and global cultures might not be practical and feasible because of other factors textbook writers have to consider. However, insufficient cultural heritage or cultural poverty for the first-language users of the textbooks used in this study may lead to cultural aphasia, a reduction in cultural identity, or the erosion of cultural sustainability.

Laine pointed out that “the sustainability of cultural education is facilitated and developed through individuals’ schooling, NGOs, local agents’ cultural sustaining activities, and school administration, as well as national educational policy and core curriculum” (Laine, 2016, p. 63). China has the largest population of EFL learners, with foreign language learning and teaching being largely achieved through the use of textbooks. The cultural landscape, therefore, may exert huge influence on learners’ cultural sustainability and the formation of their worldviews. This influence may spread beyond China because, in the context of globalization, “cultures are not separate monads but, [are] rather, heterogeneous, historically changing, interconnected” (Wierzbicka, 1997, p. 18).

The findings and discussions in this study might contribute to an awareness of cultural sustainability and the equity of intercultural communication in the compilation of textbook, as well as having implications for the further development of education policy. Furthermore, the findings may have implications for the development of EFL textbooks not only in China but also in the global education sector.

## Data availability statement

The raw data supporting the conclusions of this article will be made available by the authors. Further inquiries can be directed to the corresponding author.

## Author contributions

YL conceived of the initial idea, designed the study, drafted the manuscript, and fine-tuned the initial idea and proofread and finalized the manuscript for submission as the corresponding author. JL collected and analyzed the data. LA and YZ collected the data and revised the manuscript. All authors contributed to the article and approved the submitted version.
